# Investigation of the prevalence of impacted third molars and the effects of eruption level and angulation on caries development by panoramic radiographs

**DOI:** 10.4317/medoral.25013

**Published:** 2022-02-20

**Authors:** Handan Yıldırım, Mediha Büyükgöze-Dindar

**Affiliations:** 1 Orcid: 0000-0001-8850-3523. DDS, Asistant Professor Dr. Department of Restorative Dentistry, Faculty of Dentistry, Yeni Yüzyıl University, Istanbul, Turkey; 2 Orcid: 0000-0003-3794-4366. DDS, Asistant Professor Dr. Health Science Vocational College, Trakya University, Edirne, Turkey

## Abstract

**Background:**

This study is aimed to determine the prevalence of impacted third molars and to investigate the effects of their eruption level and angulation on caries formation in the distal of the adjacent tooth.

**Material and Methods:**

This cross-sectional study was conducted on panoramic radiographs of 38481 patients who were admitted to the Trakya University, Faculty of Dentistry. The panoramic radiographs of 7998 patients with at least one impacted third molar were included. Third molars were classified according to Winter’s classification and Pell and Gregory’s classification. The frequency of caries in partially impacted third molars and adjacent second molars was determined. The chi-square tests were used to determine potential associations between the third molars' level of eruption, angulation, and caries development.

**Results:**

The study group consisted of 4423 females (55.3%) and 3575 males (44.7%) with a mean age of 36.3±13.4 years. The prevalence of the third molar impaction rate was 23%. The impaction pattern of partially erupted third molars was characterized by an eruption level of A with the vertical position in both jaws. Partially erupted and vertically placed maxillary third molars in the level of position A caused more caries in the adjacent tooth and mesioangularly located partially erupted mandibular third molars were associated with more caries in the adjacent tooth.

**Conclusions:**

The angulation and eruption level of partially erupted third molars should be carefully examined. The prophylactic extraction of vertically and mesioangularly located third molars, especially with an eruption level of position A can be suggested to eliminate the related complications and risk of caries.

** Key words:**Dental caries, digital radiography, impacted tooth, panoramic radiography, third molar.

## Introduction

Impacted teeth are defined as those teeth that remain unerupted in the dental arch due to various systemic and local reasons within the expected time (1). The most frequently impacted teeth are undoubtedly the third molars (2) and the prevalence ranges from 16.7% to 68.6% (3). Furthermore, third molars account for 98% of all impacted teeth (4). The major etiologic factors of third molar impaction are the late maturation and lack of space. Additionally, third molars may remain impacted or semi-impacted due to reasons such as limited skeletal growth, tooth shape or position anomalies, increased regional bone density, infection, cysts, specific systemic diseases, and syndromes (5).

Every impacted third molar does not cause symptoms and pathological events. However, others can cause severe complications such as infection, atypical facial pain that can be confused with temporomandibular joint complaints, cystic lesions, and neoplasm. Besides, impacted teeth are often associated with pericoronitis, periodontitis, and detrimental effects on adjacent teeth such as bone resorption or caries (6,7). Prophylactic extraction of asymptomatic impacted molars is controversial (8) and clinical decision making regarding extraction should be based on the benefits and harms for the patient. Furthermore, the National Institute of Clinical Excellence guidelines advises against prophylactic extraction of third molars (9).

As a result of the relationship of the third molar with the second molar, the distal root surface of the second molar can be exposed to the oral environment. This relationship can be affected by the position and angulation of the third molars. Especially, partially erupted mesioangularly or horizontally positioned third molars come into contact with the distal cervical region of the second molar and increase the risk of caries development (10). The aim of this retrospective study is to determine the prevalence of impacted third molars and to investigate the effects of their eruption level and angulation on caries formation in the distal cervical region of the adjacent tooth on panoramic radiographs.

## Material and Methods

The ethical permissions required for the present study were obtained from the Scientific Research Ethics Committee of Trakya University (TÜTF-BAEK 2020/244). This cross-sectional study was planned on routinely taken panoramic radiographs of 38481 patients who were admitted to the Trakya University, Faculty of Dentistry between 2015 and 2020. The files of 38481 patients aged 25 and older were examined and panoramic radiographic images of 7998 patients with at least one impacted third molar who met the inclusion criteria were included. The inclusion criterion was the presence of the impacted third molar and the adjacent second molar. Patients with extracted third molars or the absence of adjacent second molars and who had crowns or restorations on these teeth were excluded from the study. Radiographs with superposed interdental spaces or insufficient quality for evaluation were also excluded from the study. The radiographs that were examined in this study were taken with the same panoramic x-ray device (Pax-Flex 3D Vatech, Hwaseong, South Korea) at Trakya University, and none of the radiographs were exposed especially for this study.

**Figure 1:**
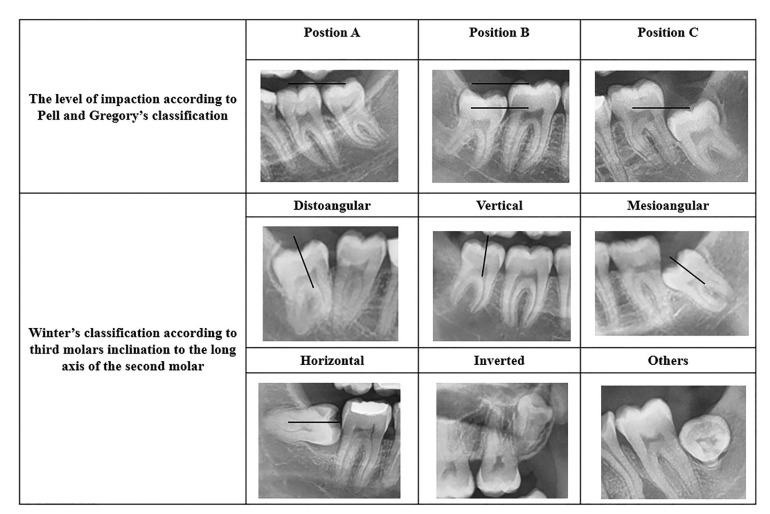
Radiographic images of the Pell and Gregory Classification and the Winter classification.

The radiographic images were evaluated by the same observer on the computer monitor with subdued ambient lighting and third molars were classified according to the eruption status (fully erupted, partially erupted, or unerupted), angulation based on Winter’s classification, and the level of impaction according to Pell and Gregory’s classification (Fig. 1). In the Winter classification, the angulation of the impacted tooth is determined by measuring the angle between the long axis of the tooth and the occlusal plane on panoramic radiography. According to this classification, third molars were grouped as distoangular (>90°), vertical (90°- 61°), mesioangular (60°- 31°), horizontal (30°- 0°), inverted (0°), and others (11). According to the Pell and Gregory classification, third molars were defined according to their relationship with the occlusal plane of the second molar. Accordingly, three positions are specified (12). Position A: the highest part of the impacted third molar is at or above the occlusal plane. Position B: the highest part of the impacted third molar is below the occlusal plane but above the cementoenamel junction (CEJ) of the second molar. Position C: The highest part of the impacted third molar is below the CEJ of the second molar. The classification of the pathologic conditions was determined on panoramic radiographs and classified as follows: 1) distal caries on the second molar; 2) mesial caries on impacted third molar; 3) caries on both third and second molars; 4) no caries.

Statistical analysis was performed with IBM SPSS Statistics for Windows, Version 23.0 (SPSS Inc., Chicago, IL, USA). Descriptive statistics such as prevalence, frequency distributions, and percentages were calculated for the gender, and classification of third molars' position, and angulation. The chi-square tests were used to determine potential associations between the third molars' position, angulation, and caries development. Any *P* value less than 0.05 was considered significant.

## Results

Of the 38481 patients aged 25 years and older included in the study, 8828 had at least one impacted third molar tooth. The prevalence of impaction rate was 23%. Since the quality of panoramic radiographs of 830 patients was not suiTable for radiological evaluation, the study group consisted of radiographs of 7998 patients. Distributions of impacted third molars were given in Table 1. 55.3% of the impacted third molars were observed in females and 44.7% in males. There was a significant difference between the genders (*p*=0.00) and impacted third molars were observed more frequently in females. When the locations of the impacted third molars were examined, a significant difference was observed between the jaws (*p*=0.012). However, no statistically significant difference was found between the right and left sides.

Since 98.4% of impacted third molars in the maxilla and 70% in the mandible are fully unerupted, their effects on the adjacent tooth were not evaluated. Of the fully unerupted third molars in the maxilla, 56.6% were in vertical, 27.2% distoangular, 11.1% mesioangular, 1.7% horizontal, and 0.3% inverted position. On the other hand, 58.2% of the fully impacted third molars in the mandible were in mesioangular, 16.7% horizontal, 16.3% vertical, 6.6% distoangular, and 0.01% inverted position, and there was a significant difference between jaws and fully unerupted third molars’ angulations (*p*=0.000). While most of the fully unerupted maxillary third molars were in the vertical position, the mesioangular position was more common in the mandibular third molars.

The level of impaction and angulations of the partially erupted third molars were shown in Table 2. A statistically significant relationship between the level of impaction and angulations of the partially erupted third molars was observed in both the maxilla and mandible. Partially erupted third molars were mostly in vertical status and position A in both maxilla and mandible.

Of the partially erupted maxillary third molars, 65.6% were associated with caries either in the impacted third molar or in the adjacent tooth. Caries was present in 33.6% of the partially erupted maxillary third molars. 19.7% of the partially erupted maxillary third molars have caused distal caries in the adjacent tooth. On the other hand, 12.3% of partially erupted maxillary third molars have caused caries both in themselves and in the adjacent teeth. When the relationship between the degree of eruption of the partially erupted maxillary third molars and caries was examined, a significant relationship was observed (Table 3). The partially erupted maxillary third molars with an eruption level of position B caused caries more frequently both in themselves and in the adjacent teeth. Similarly, the angulation of the partially erupted maxillary third molars was also effective on caries formation. The partially erupted maxillary third molars, which most frequently cause caries in the adjacent tooth, were vertically located followed by the mesioangular position. The presence of caries in the vertically partially erupted maxillary third molars with eruption level of position A was statistically significantly higher than in other positions (*p*=0.040).

**Figure 2:**
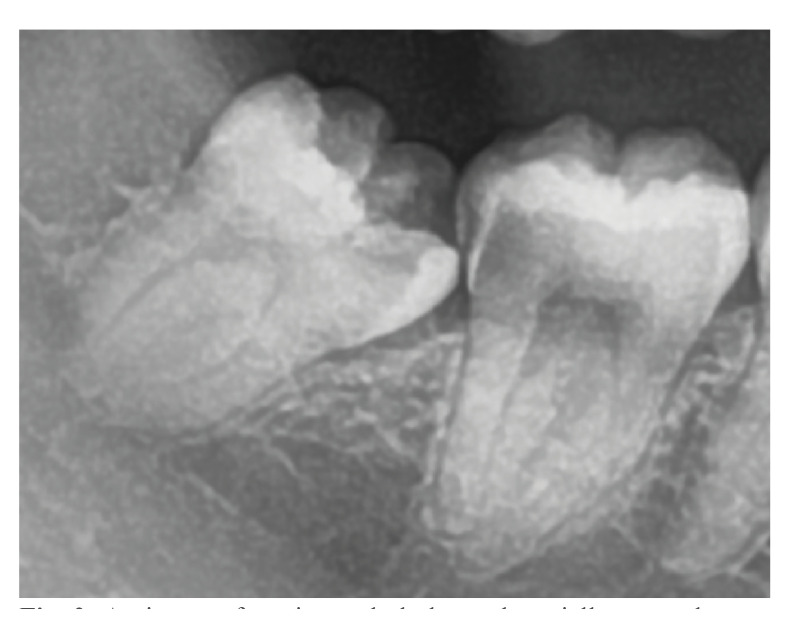
An image of mesioangularly located partially erupted mandibular third molar with an eruption level of position A and the presence of caries in the third molar and adjacent tooth.

Of the partially erupted third molars in the mandible, 18.7% caused caries in the adjacent teeth, 4.2% in themselves, and 13.4% in both. Partially erupted mandibular third molars in position A and the mesioangular location were associated with significantly more adjacent tooth caries than other positions (Fig. 2).

**Table 1:**
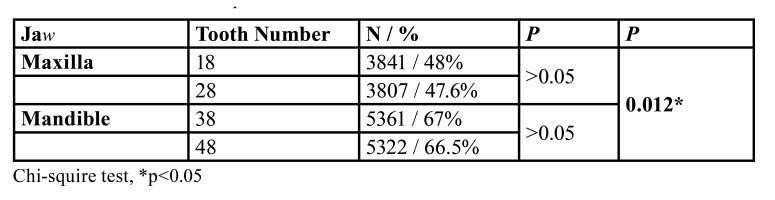
Distributions of impacted third molars.

**Table 2:**
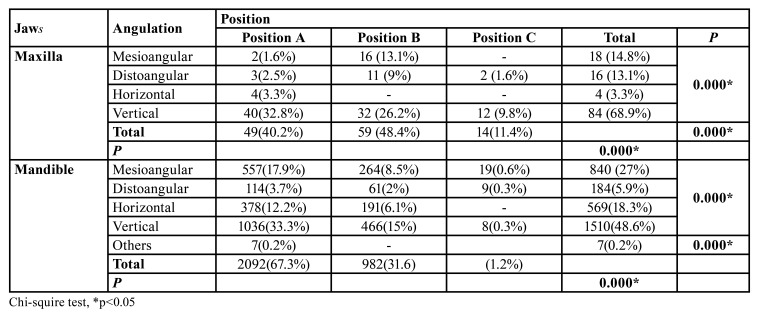
Distributions of the positions and angulations of the partially erupted third molars.

**Table 3:**
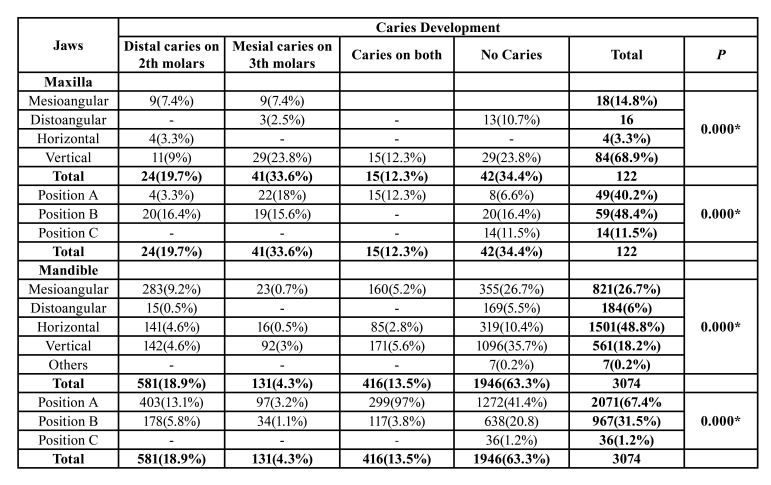
The relationship between the degree eruption level and angulation of partially erupted third molars with caries formation.

## Discussion

The present study investigated the prevalence of impacted third molars and the associations between their level of eruption and angulation on caries formation in the distal cervical region of the adjacent tooth on panoramic radiographs. The reported prevalences of impacted third molars vary in different racial and ethnic groups in previous studies (13-15). Therefore, it was planned to investigate the prevalence and associations in a large sample of Turkish patients in the Trakya Region. Although there have been several studies investigating the prevalence of impacted third molars and the associations between their position and angulation (16,17), only a few studies focused on detrimental effects of impacted third molars on the adjacent tooth (18-20) and almost none have been conducted with such a large sample.

The reported prevalence of impacted third molars was 68.6% by Quek *et al*. (21), 52.3% by Jain *et al*. (13), and 35,9% by Celikoğlu *et al*. (22). In the present study, the prevalence rate was 23% which is very similar to the rate of 21.9% reported by Ventä *et al*. (16). These conflicting prevalence rates can be explained primarily by racial differences and sampling techniques. The diagnostic methods and criteria could likely affect the prevalences. The higher prevalence rates were reported, when the study investigated only the mandibular third molars. Besides, higher prevalence rates reported in most studies were related to the sample including the age of 18 or 20 (17,19). However, it has been stated in various studies that the third molar eruption can continue until the age of 25 (23). The reason for the lower prevalence observed in the present study compared to previous studies was caused by the inclusion of individuals aged 25 and older. Ventä *et al*. reported a lower rate than the present study by including individuals over 30 years of age (16). The diagnostic method used also had an effect on prevalence. Most impacted third molars cannot be detected by clinical examination alone without radiographic evaluation.

Although some studies reported that there was no difference between gender and the prevalence of impacted third molars (16,24), most studies, including the present study, stated that the frequency of impacted third molars was significantly higher in females (25). The higher prevalence rates of impacted third molars in females can be attributed to the smaller size of the jaw compared to males and the lack of space for eruption.

Regarding the distribution of impacted third molars according to the jaws, the present study showed that frequency in the mandible (66,5%) was significantly higher than in the maxilla (47.8%). This finding was consistent with Kumar Pillai *et al*. (26). Additionally, Hashemipour *et al*. reported 1.9 times higher incidence of third molar impaction in the mandible (3). Furthermore, many studies including the present study observed that vertical angulation is most common in both the maxilla and the mandible (11,26), while a few reported that mesioangular impaction was the most common pattern of angulation (17,24).

According to Pell and Gregory’s classification, the most reported eruption level of partially erupted third molars in the present study was position B in the maxilla, position A in the mandible. On the other hand, Quek *et al*. (21) and Hassan (27) stated the B level was the most common level of impaction in both jaws. However, these studies did not classify impacted third molars such as fully impacted or partially erupted. Moreover, consistent with our study, Kumar *et al*. (2) reported that the most common eruption level in the mandible is position A.

Considering the results of this study, the presence of distal caries in the second molar is 19.7% for the mandible, 18.7% for maxilla, consistent with the rate of 14.74% reported by Claudia *et al*. (1), and 21.5% by Al-Khateeb and Bataineh (28). However, the reported rate in the present study was higher than 12.6% reported by Polat *et al*. (29) and 2.5% reported by Sejfija *et al*. (25). Furthermore, caries was present in 33.6% of the partially erupted maxillary and 4.2% in mandibular third molars. A similar rate of 5.3% was reported for mandibular third molars by Polat *et al*. (29). Both the level of impaction and angulations of partially impacted third molars were risk factors for distal cervical caries on adjacent teeth. Vertically located maxillary impacted third molars with an eruption level of position B were related with distal caries on second molars while mesioangularly located mandibular molars with position A mostly related with caries. There were studies in the literature which supported the high risk of distal caries on second molars associated with mesioangularly or horizontally located mandibular third molars (29-31). However, the number of studies on maxillary impacted third molars was quite limited. In a study conducted by Khawaga *et al*. (32), it was stated that the caries incidence was higher in vertical angulation of the tooth numbered 28. This study supported the findings of the present study. The main reason for the development of cervical caries on the adjacent tooth of the mesioangularly positioned mandibular molar was that they cause an unaccessible contact area between two teeth and consequently proper cleaning becomes impossible (1). On the other hand, even vertically positioned maxillary third molars can cause cavities due to the difficulty of oral hygiene procedures and the inaccessibility of the toothbrush in the maxilla. Therefore, all third molars in abnormal positions should be followed regularly, and extraction should be considered as a treatment option if necessary to avoid complications such as caries.

Within the limitations of this study, demineralization and initial enamel caries in third molars and adjacent teeth could not be evaluated. Another limitation of the present study was that only panoramic radiographs were used. The most reliable way for caries diagnosis was the combination of both clinical and radiographic examination. Even though, bite-wing radiographs are the golden standard for approximal evaluation, routinely taken panoramic radiographs can also be used. In addition, a larger sample group can be reached by examining routinely taken panoramic radiographs, and more reliable results can be obtained with a larger sample. Besides, patients were prevented from being exposed to extra doses of radiation. However, detailed evaluations can be made and more accurate estimates of the risk of complications can de determined by using cone beam computerized tomography (CBCT) scans (33).

## Conclusions

The pattern of partially erupted third molar impaction was characterized by a high prevalence rate of level A impaction with the vertical position in both jaws. Partially erupted and vertically placed maxillary third molars in the level of position A caused more caries in the adjacent tooth. On the other hand, mesioangularly located partially erupted mandibular third molars were associated with more caries in the adjacent tooth.

In conclusion, the angulation and eruption level of partially erupted third molars should be taken into consideration in terms of complications such as caries. The prophylactic removal of vertically and mesioangularly located third molars, especially with an eruption level of position A can be suggested.
